# Unequal Burdens: Gendered and Socioeconomic Dimensions of Occupational Health Among Hong Kong’s Informal Waste Pickers

**DOI:** 10.3390/healthcare13060683

**Published:** 2025-03-20

**Authors:** Siu-Ming Chan, Yuen-Ki Tang, Heng Xu, Jasmine Zhang, Kim Kwok, Wai-Yiu Tam, Wing-Him Tang

**Affiliations:** 1 Department of Social and Behavioural Sciences, City University of Hong Kong, Hong Kong SAR, China; yuenki.tang@cityu.edu.hk (Y.-K.T.); hengxu4@cityu.edu.hk (H.X.);; 2 Waste Picker Platform, Mission to New Arrivals, Hong Kong SAR, China

**Keywords:** informal waste pickers, gender, socioeconomic status, working condition, occupational income, mental health, Hong Kong

## Abstract

**Background**: This study explores the multifaceted inequalities faced by informal waste pickers in Hong Kong, focusing on the impacts of gender and socioeconomic status in shaping their working environment, income, and psychological health. Recognizing that social stratification encompasses a series of structural factors, i.e., gender, race, and socioeconomic status, we aim to fill the gap in existing literature regarding the precarious employment of this population. **Methods**: Utilizing a comprehensive, territory-wide survey, we analyzed the experiences of male and female waste pickers across different socioeconomic backgrounds. Differences between genders for continuous variables were assessed using the independent samples *t*-test. Differences across categories defined by gender and socioeconomic status were analyzed using one-way ANOVA, followed by post hoc comparisons with the least significant difference (LSD) method. **Results**: Our findings indicate that both gender and socioeconomic status significantly influence working environment, occupational income, and psychological health outcomes. Specifically, female waste pickers from lower socioeconomic backgrounds face more adverse working environments, lower income levels, and heightened psychological health risks compared to their higher-status counterparts. **Conclusions**: These results underscore the urgent need for targeted outreach and tailored healthcare services for vulnerable female waste pickers, as well as social support systems that empower them to negotiate with intermediaries and recycling shop owners. By recognizing their essential role in Hong Kong’s urban recycling ecosystem, this study advocates for policies that address these disparities and promote psychological health and social well-being among this marginalized group.

## 1. Introduction

Precarious employment, marked by significantly high job insecurity, lack of workplace protections, susceptibility to unfair treatment, and financial instability, is strongly associated with low wages, economic challenges, and negative self-assessments of mental health [1,2,3,4]. Although the entire precarious employment population faces numerous challenges, females in such roles are found to have significantly higher odds of reporting poor mental health [5]. Informal waste pickers, a group that encompasses around 15 million individuals worldwide, are involved in recycling materials such as metals, glass, plastic, and paper. They represent a significant segment of the precariously employed workforce [6]. Informal waste pickers are often overlooked as key contributors to urban society, despite their critical role in the recycling industry and promoting sustainable development [7]. They are frequently marginalized from formal waste management systems and face significant occupational hazards, such as exposure to toxic substances, excessive noise, and extreme temperatures [8]. Meanwhile, they are found to be disadvantaged in price bargaining and to be vulnerable to the exploitation from intermediaries [9]. Although previous literature has identified a series of risk factors that undermine informal waste pickers’ health and decrease their occupational income, there are limited efforts in investigating the health and occupational income inequalities among the informal waste picker population across gender and other forms of social stratification, i.e., socioeconomic status. The 17 Goals of Sustainable Development initiated by the United Nations Department of Economic and Social Affairs highlight the necessities and urgency of ensuring healthy lives and well-being for all, achieving gender equity and empowering females, and promoting full, productive employment and decent work for all [10]. Our study aims to examine the occupational income and mental health disparities faced by informal waste pickers in Hong Kong, with a particular focus on female workers. It investigates the gender differences in income and mental health outcomes within this vulnerable population. The purpose of the study is to provide insights into developing interventions that can improve mental health and promote decent working conditions for people in the waste-picking industry.

Various forms of social stratifications, such as race, gender, class, and other identities, are documented, as the structural forces contribute to the unique experiences of oppression and privilege for individuals and groups. By investigating the disadvantages arising out of gender and other oppressive institutions, i.e., class and race, public health researchers have highlighted how health inequalities are shaped by multiple axes of social division [11,12]. Health disparities driven by socioeconomic status are evident through shorter life expectancy and higher rates of illness and death among those with limited education, lower incomes, and less prestigious occupations [13,14]. Likewise, gender also plays a crucial role as a social determinant, shaping the access to resources that individuals need to achieve their best possible health [15].

Therefore, this study will investigate the occupational income and mental health inequalities among informal waste pickers in Hong Kong across gender and socioeconomic status.

### The Context in Hong Kong

In Hong Kong, the high cost of living and eroding intergenerational support in low-income households exacerbate financial precarity among older adults, compelling many to remain economically active despite systemic age discrimination and limited formal employment opportunities in the local job market. Between 2008 and 2018, the employed population aged 65+ surged from 42,600 to 139,300, with labor force participation more than doubling to 11.7% [16]. By 2022, 42.3% of employed older adults worked in low-wage, physically demanding sectors—such as cleaning, security, and waste picking—compared to 27.8% of the general workforce [17]. Fieldwork highlights the prevalence of informal labor, particularly waste picking, which remains unaccounted for in official census data and lacks labor protections. This institutional invisibility perpetuates vulnerabilities, as elderly informal workers lack access to workplace safeguards. Informal waste pickers are informal because their work is unregulated and not officially recognized. They operate outside formal employment systems without contracts, legal protections, or benefits, relying on self-initiated activities like collecting and selling recyclables. This informality stems from limited job opportunities, social marginalization, and a lack of inclusive policies. Regarding the systemic inequities faced by the elderly in the waste picking industries, they are categorized as informal waste pickers.

A survey conducted in 2018 by the Waste Pickers Platform, which included 505 informal waste pickers, revealed that most of the participants were aged over 60. On average, they earned approximately HK$716 (around USD 91) per month through recycling activities [18]. The survey findings revealed that the majority of informal waste pickers participate in waste collection to sustain a basic livelihood and meet their daily needs. They often face a hostile social environment, such as theft of personal belongings and recyclable materials, and lack access to social insurance coverage [19]. Although informal waste pickers make significant contributions to local waste management and sustainable development, their efforts often go unacknowledged. Recent studies highlight that they face numerous occupational risks and hazards, such as exposure to toxic substances and traffic accidents, which are frequently linked to poor health outcomes. Additionally, they remain vulnerable to punitive actions by authorities, including fines and the confiscation of their belongings [20,21].

## 2. Methods

### 2.1. Data and Sample

The data for this study were sourced from the Hong Kong Waste Pickers Research Report 2023, a collaborative project conducted by various academic institutions and NGOs. Ethical approval for the study was obtained from the City University of Hong Kong’s Human Subjects Ethics Sub-Committee (Ref: HU-STA-00000994). Informed consent was secured directly in street-based settings, where researchers explained the study details comprehensively and ensured participants’ understanding before obtaining their voluntary agreement. Data were collected through face-to-face questionnaires at 84 recycling shops and 11 recycling trolleys across all 18 districts. Collection periods were scheduled from 9:00 AM to 4:00 PM daily, from 18 November to 24 November 2023. A total of 320 volunteer instructors handled operational management and surveys, organized into teams of two to three interviewers at each site, ensuring efficient data gathering. The survey period concluded with 914 engaged respondents. Inclusion criteria encompassed both male and female waste pickers active on streets or in buildings. The exclusion criterion was individuals who store collected waste at home. Respondents were initially divided into two categories based on their recycling practices: those who recycled at home and those who engaged in recycling activities within community streets or buildings. The analysis specifically targeted the latter group, concentrating on individuals involved in community recycling activities. After these exclusions of home recycling cases and those who provided inadequate responses, analysis proceeded with a sample of 701 valid questionnaires. Details of the sampling procedure for the survey have been documented in a previous study [22], which utilized the same dataset as this work.

### 2.2. Measurement

Demographic variables. Demographic characteristics of the respondents, such as sex and age, were incorporated into the analysis.

Socioeconomic variables. Individual monthly income was used to assess socioeconomic status, with the latest Hong Kong poverty line serving as the classification standard [23].

Working condition and occupational income of informal waste picking. Respondents were asked about their total working time (years) as informal waste pickers, working day/week, working turn/day, working hours/day, sale recycling weight per turn, money earned from recycling sale per turn, money earned from recycling sale per month, and ratio of monthly recycling sale income to total monthly income.

Mental health variables. Mental health variables were assessed using abbreviated forms of the Patient Health Questionnaire (PHQ) and General Anxiety Disorder (GAD) scales due to the street-based nature of the survey and the time constraints involved in data collection. The two-item versions, PHQ-2 and GAD-2, served as the instruments for this study. Both PHQ-2 and GAD-2 function as screening tools to detect potential signs of depression and anxiety, but they do not provide clinical diagnoses. These tools represent the dependent variables in the research. Comprising the first two items of the full PHQ-9 depression scale, PHQ-2 includes a stem question that asks, “Over the last two weeks, how often have you been bothered by any of the following problems”? The associated items are “Little interest or pleasure in doing things” and “Feeling down, depressed, or hopeless”. Responses are rated on a Likert scale from 0 (not at all) to 3 (nearly every day), with total scores ranging from 0 to 6. Validation studies have confirmed the high sensitivity of the PHQ measurement in detecting major depression [24,25]. The GAD scale adopts a similar stem question and scoring system as the PHQ but includes the items “Feeling nervous, anxious or on edge” and “Not being able to stop or control worrying”. Scores of 3 or higher on the PHQ-2 and GAD-2 are recommended for identifying major depressive disorder and generalized anxiety disorders, respectively [25,26,27]. Previous research has validated the Chinese versions of the PHQ-2 and GAD-2 within the Chinese population [28,29]. In this study, the internal consistency reliability for the PHQ-2 was 0.814, and for the GAD-2, it was 0.837.

### 2.3. Statistical Analysis

Statistical analyses were conducted to examine the relationships and differences in working conditions, occupational income, and mental health among informal waste pickers, considering variables such as gender and socioeconomic status. For continuous variables, differences between genders were assessed using the independent samples *t*-test. Further, differences across categories defined by both gender and socioeconomic status were analyzed using one-way ANOVA, followed by post hoc comparisons using the least significant difference (LSD) method.

## 3. Results

### 3.1. Demographic

The demographic characteristics of the study participants are presented in Table 1. The age distribution indicates a majority of participants aged between 60 and 69 years (37.9%), followed by those aged 70–79 years (35.5%). The smallest age group represented is those 50 years or under, comprising only 4.3% of the sample. Regarding gender distribution, a significant majority of the participants are female (81.4%), while males represent 18.6% of the sample. As presented in Table 2, the average income earned from informal waste picking activities per month reported by participants is approximately $1431.16, and the average income per month is reported as $5826.68. The mean scores of the PHQ-2 and GAD-2 are 0.82 and 0.86, respectively. In our study, we utilized the GAD-2 and PHQ-2 scales to assess anxiety and depression among participants, applying a cutoff score of 3. For the PHQ-2, scores below 3 were recorded for 115 males (19.7%) and 468 females (80.3%), while scores of 3 or above were seen in 8 males (10.8%) and 66 females (89.2%). Regarding the GAD-2, scores below 3 were observed in 115 males (19.8%) and 467 females (80.2%), and scores of 3 or above were observed in 7 males (9.2%) and 69 females (90.8%).

### 3.2. Comparative Results

In the exploration of gender differences across various work-related metrics (Table 3), several significant findings emerged. For the variable “working hours per day”, males reported a mean of 5.96 h, significantly higher than the female mean of 5.10 h (t = 2.159, *p* = 0.031). Similarly, the “sale weight per turn” was significantly greater for males, with a mean of 36.87 compared to 28.68 for females (t = 2.602, *p* = 0.01). Moreover, males exhibited a higher mean “money earned from recycling sale per turn” at 48.01, distinctly surpassing the female mean of 32.57 (t = 2.758, *p* = 0.007). Furthermore, the “money earned from recycling sale per month” also displayed notable gender disparities, with males achieving a higher mean of 1805.65 compared to 1341.79 for females (t = 2.27, *p* = 0.024). For the ratio of monthly recycling sale money over monthly income, the males mean percentage is 37%, which is significantly lower (*p* = 0.045) than that for females (31%). In addition to the primary analyses, effect sizes were calculated to further understand the impact magnitude of the observed differences. The computed effect sizes ranged from 0.058 to 0.398.

As Table 4 illustrates, the analysis of work-related variables, stratified by gender and socioeconomic status levels, uncovered significant disparities across several metrics. Regarding “sale weight per turn”, a significant difference was noted between females earning below $5000, who had a mean of 24.29 (SD = 24.07), and males earning over $5000, who recorded a mean of 42.09 (SD = 31.07), *p* < 0.001. Meanwhile, there is a significant difference between females earning below $5000 and their female counterparts earning $5000 above, with a mean of 35.04 (SD = 28.6). In the metric of “money earned from recycling sale per turn”, females earning less than $5000 demonstrated a significantly lower mean of 28.16 (SD = 24.27) compared to males earning $5000 above, whose mean was 53.78 (SD = 50.37), *p* < 0.001. Moreover, they demonstrated a significantly lower mean compared to their female counterparts earning $5000 above, whose mean was 39.09 (SD = 32.18). In terms of “money earned from recycling sale per month”, females earning less than $5000 showed a significantly lower mean of 991.67 (SD = 1063.33) compared to males earning $5000 above, whose mean is $2248.31 (SD = 2402.38), and their female counterparts earning $5000 above, whose mean is 1800.63 (SD = 2749.64). Effect size results ranged from 0.015 to 0.09.

Table 5 demonstrates the mental health analysis results between genders. For PHQ-2, the score of females (0.91 ± 1.53) was significantly higher than males (0.65 ± 1.22), *p* = 0.041 < 0.05. Similarly, as for the GAD score, the males (0.56 ± 1.34) were also lower than females (0.88 ± 1.57), with *p*-value = 0.011 < 0.05. The effect size result of the PHQ-2 is 0.176; for the GAD, it is 0.209.

Table 6 presents the comparative results of mental health, assessed using the PHQ-2 and GAD-2 scales, across different genders and socioeconomic groups. There is no significant difference between subgroups in terms of self-reported depression measured by the PHQ-2. Concerning self-reported anxiety measured by the GAD-2, females whose earnings were below $5000 reported a significantly higher level of anxiety (mean = 1.07, SD = 1.73) compared to males earning below $5000 (mean = 0.54, SD = 1.26) and males earning above $5000 (mean = 0.63, 1.47). Meanwhile, their mean score of anxiety is significantly higher than that of their female counterparts earning $5000 above (mean = 0.69, SD = 1.35). The effect size result of the PHQ-2 is 0.08; for the GAD, it is 0.018.

## 4. Discussion

Examining our results through a stratification perspective uncovers a broader range of inequalities compared to traditional binary methods [30]. This approach also enables us to assess the position of female waste pickers with lower socioeconomic status relative to others and to measure the disparity between those who are most advantaged and those who are least advantaged. Nevertheless, the reliance on a simple stratification approach has methodological limitations. While this approach effectively identifies general trends and disparities, it may oversimplify the complexity of intersecting inequalities among subgroups. In line with previous literature, which reflects that females are associated with increased probability of reporting disadvantaged working conditions and negative health outcomes [5], our study showed that female respondents are worse off than male counterparts, especially those with higher socioeconomic status, in terms of income from waste picking activities, including income earned per turn and monthly income. As our findings reveal that female aged above 60 make up the majority of respondents, we further explain that female respondents, especially elderly, are less capable of carrying the heavier workloads and working longer than their male counterparts. This is supported by the findings that female respondents have less sale weight per turn and less working hours per day than their male counterparts. Echoing a previous study that highlights elderly female informal waste pickers’ disadvantaged position in front of the recycling industry with regard to the recyclable price [21], we explain that the female background may undermine respondents’ position when negotiating with an intermediary and further limit the possibility of earning more money by waste picking. The results of both the GAD-2 and PHQ-2 show that female informal waste pickers have a higher risk of expressing depression and anxiety than male informal waste pickers. Our study findings offer evidence for the fact that gender still plays a crucial role in the emergence of complex occupational and health inequalities in informal employment sectors in the Hong Kong context.

Additionally, the findings of our study highlight “internal” inequalities within the female informal waste picker group. First, it is striking that a wide gap for self-reported anxiety is observed between females earning below $5000 and those earning $5000 or above. These findings may be associated with the fact that horizontal and vertical segregation puts female informal waste pickers with lower socioeconomic status in a doubly disadvantaged position with less resources and support to counter the adversities and hazards in waste picking activities. Second, female informal waste pickers with lower socioeconomic status have been found to earn less money per turn and per month from waste picking activities than their female counterparts with higher socioeconomic status. We explain the observed income gap from two perspectives. On the one hand, female respondents with lower socioeconomic status are found with worse mental health conditions, which may further undermine their capacities to undertake heavy waste picking workloads. On the other hand, female respondents with lower socioeconomic status may lack the skills and necessary supports to bargain with intermediaries or recycling shop owners for more acceptable prices and claim rights as indispensable actors in the Hong Kong urban recycling system.

Taken together, gender and socioeconomic status appear to have implications for informal waste pickers’ working conditions and physical and mental health in Hong Kong. Understanding the roles of these forms of social stratification, although complex and multifactorial, may help to guide the development of strategies for overcoming the perceived barriers that female informal waste pickers encounter in and beyond waste picking activities. First, the inequalities in income from waste picking were predominantly associated with gender bias, which refers to female waste pickers’ relatively disadvantaged position in price bargaining with intermediaries and thus disproportionately affected female informal waste pickers. Ameliorating the gender inequality in waste picking income would likely necessitate more gender-focused strategies. Second, our study findings highlight that female informal waste pickers with lower socioeconomic status earn less income from waste picking and have worse mental health conditions than other subgroups. Therefore, a more holistic intervention to ameliorate the diversities and challenges encountered by females with lower socioeconomic status is necessary.

First, strong cooperation networks must be established among social service organizations, government, and recycling industries to ensure the basic rights and interests of female informal waste pickers, especially those with lower socioeconomic status. Social service organizations or social workers should provide necessary protection services, i.e., plastic gloves and legal aids to prevent female informal waste pickers from hazardous working conditions and exploitation from recycling industries. Second, social work organizations should cooperate with health/medical professionals to develop and offer more tailored mental health services to female informal waste pickers with lower socioeconomic status to counter the risks and adversities that may lead to anxiety. Third, some aging people have taken up waste picking to make ends meet due to insufficient retirement protection in Hong Kong. The government should review how to increase the level of retirement protection for the elderly as a whole, including the establishment of a universal aging pension system, so as to ensure that the elderly can have a stable retirement life rather than continuing to engage in high-risk informal work.

Income and health inequalities associated with gender and socioeconomic status exist and appear to have implications for informal waste pickers in Hong Kong. Our analysis highlighted that female informal waste pickers with lower socioeconomic status represent a particularly vulnerable group, as their challenges seem to be amplified by multiple layers of inequality. We believe the findings of this study can help shed light on how the diversity within the informal waste picker community is linked to various forms of social stratification. Future studies are warranted to evaluate working and health conditions across other informal waste picker cohorts and to evaluate their implications for developing more tailored interventions.

This study has several limitations that should be considered. The survey was conducted within a single week, which may not have been sufficient to capture the full range of waste pickers in Hong Kong, thereby limiting the generalizability of the findings. The use of a brief street-based questionnaire, while efficient for data collection, lacked the depth and nuance that could have been achieved through more extensive methods, such as in-depth interviews. Additionally, the study employed a simple methodological approach grounded in a stratification perspective, which effectively highlighted broad patterns of inequality but may lack the granularity to fully explore the complexity of intersecting social factors. Furthermore, questions related to socioeconomic status and mental health relied on self-reported data, which are subject to biases such as inaccuracies or social desirability effects. Additionally, some participants were actively working while responding to the survey, which may have constrained their ability to provide detailed and thoughtful answers. Future research should adopt a more comprehensive approach to address these limitations.

## 5. Conclusions

The present study demonstrated the significant impact of gender and socioeconomic status on the working conditions, income, and mental health of informal waste pickers in Hong Kong. Our findings reveal that female waste pickers, particularly those with lower socioeconomic status, face compounded disadvantages that affect their ability to earn a sustainable income and maintain good health. The disparities highlighted in this research call for targeted interventions that address both gender and socioeconomic inequalities faced by informal waste picker population. To mitigate these challenges, it is essential to establish comprehensive cooperation networks among social service organizations, government bodies, and the recycling industry. Providing protective services, legal aid, and outreached, tailored mental health support can help improve the working conditions and overall well-being of female informal waste pickers. Meanwhile, enhancing broader retirement protection for the elderly can reduce their reliance on high-risk informal occupations. Future research should aim to capture a more comprehensive picture of the informal waste picker community by employing more extensive data collection methods and considering a broader range of participants. It will provide valuable insights into supporting this vulnerable population and promote greater equity within the informal employment sector.

## Figures and Tables

**Table 1:** Demographic results.

		N	%
Age	50 or under	19	4.3
	50–59	80	11.8
	60–69	256	37.9
	70–79	249	35.5
	80 or above	69	10.2
Gender	Female	550	81.4
	Male	126	18.6

**Table 2:** Income and mental health results.

	N	Mean	SD
Income earned from informal waste picking/month	642	1415.81	2009.40
Total income/month	641	5880.88	3984.33
GAD-2	658	0.82	1.54
PHQ-2	657	0.86	1.48

**Table 3:** Comparative results of working conditions between genders.

	Male			Female			t	*p*	Cohen’s d
	N	M	SD	N	M	SD			
total working time (years)	47	8.64	7.14	261	10.13	10.12	−0.970	0.333	0.155
working day/week	125	5.87	1.65	528	5.77	1.77	0.566	0.572	0.058
working turn/day	119	3.02	2.98	530	2.54	2.34	1.898	0.058	0.191
working hours/day	121	5.96	4.20	519	5.10	3.88	2.159	0.031	0.217
sale weight/turn	101	36.87	29.14	460	28.68	26.47	2.602	0.010	0.301
sale money/turn	122	48.01	60.34	534	32.57	28.26	2.758	0.007	0.398
sale money/month	118	1757.39	1925.10	511	1333.31	2023.11	2.071	0.019	0.212
monthly sale money/monthly income	109	0.37	0.32	478	0.31	0.34	1.701	0.045	0.179

**Table 4:** Comparative results of working conditions across gender and socioeconomic status.

	Male						Female						F	*p*	η^2^
	<5000			>5000			<5000			>5000					
	N	M ± SD	95%CI	N	M ± SD	95%CI	N	M ± SD	95%CI	N	M ± SD	95%CI			
total working time (years)	22	8.68 ± 5.93	[6.04–11.32]	25	8.60 ± 5.93	[5.22–11.97]	136	9.54 ± 10.81	[7.70–11.37]	113	11.13 ± 9.5	[9.36–12.90]	0.899	0.442	0.09
working day/week	56	6.11 ± 1.61	[5.68–6.54]	62	5.68 ± 1.72	[5.24–6.12]	277	5.75 ± 1.81	[5.53–5.96]	232	5.78 ± 1.75	[5.55–6.00]	0.764	0.515	0.04
working turn/day	52	2.98 ± 3.09	[2.11–3.84]	60	3.13 ± 3.03 ^	[2.34–3.91]	279	2.56 ± 2.33	[2.28–2.83]	231	2.44 ± 2.11 #	[2.17–2.72]	1.749	0.156	0.08
working hours/day	55	6.25 ± 4.41 ^	[5.06–7.44]	60	5.89 ± 4.13 ^	[4.82–6.96]	275	5.42 ± 4.01	[4.94–5.90]	224	4.73 ± 3.71 *#	[4.24–5.22]	3.127	0.025	0.015
recycling sale weight/turn (kg)	43	32.25 ± 26.54	[24.08–40.42]	52	42.09 ± 31.0 ×	[33.44–50.74]	246	24.29 ± 24.07 ^#	[21.26–27.31]	199	35.04 ± 28.6 ×	[31.04–39.04]	9.663	<0.001	0.051
money from recycling sale/turn	55	34.92 ± 36.16 #	[25.14–44.69]	60	53.78 ± 50.37 *×^	[40.76–66.78]	287	28.16 ± 24.27 #^	[25.34–30.98]	228	39.09 ± 32.18 #×	[34.88–43.28]	12.834	<0.001	0.058
money from recycling sale/month	52	1304.42 ± 1089.1 #	[1001.21–1607.63]	59	2248.31 ± 2402.38 *×	[1622.24–2874.36]	272	991.67 ± 1063.33 #^	[1437.76–2163.49]	223	1800.63 ± 2749.64 ×	[1276.26–1600.81]	10.385	<0.001	0.049

* *p* < 0.05 compared to male < 5000, # *p* < 0.05 compared to male > 5000, × *p* < 0.05 compared to female <5000, ^ *p* < 0.05 compared to female > 5000.

**Table 5:** Comparative results of mental health between female and male informal waste pickers.

	Male			Female			t	*p*	Cohen’s d
	N	M ± SD	95%CI	N	M ± SD	95%CI			
PHQ-2	123	0.65 ± 1.22	[0.43–0.86]	534	0.91 ± 1.53	[0.78–1.04]	−2.056	0.041	0.176
GAD-2	122	0.56 ± 1.34	[0.32–0.80]	536	0.88 ± 1.57	[0.71–0.94]	−2.320	0.011	0.209

**Table 6:** Comparative results of mental health across gender and socioeconomic status.

	Male						Female						F	*p*	η^2^
	<5000			>5000			<5000			>5000					
	N	M ± SD	95%CI	N	M ± SD	95%CI	N	M ± SD	95%CI	N	M ± SD	95%CI			
Depression (PHQ-2)	57	0.71 ± 1.16	[0.41–1.02]	61	0.60 ± 1.32	[0.26–0.94]	288	1.01 ± 1.64	[0.81–1.20]	229	0.82 ± 1.40	[0.64–1.01]	1.723	0.161	0.08
Anxiety (GAD-2)	57	0.54 ± 1.26 ×	[0.25–0.88]	60	0.63 ± 1.47 ×	[0.87–1.27]	289	1.07 ± 1.73 *#^	[0.51–0.87]	230	0.69 ± 1.35 ×	[0.51–0.87]	3.961	0.008	0.018

* *p* < 0.05 compared to male < 5000, # *p* < 0.05 compared to male >5000, × *p* < 0.05 compared to female <5000, ^ *p* < 0.05 compared to female > 5000.

## Data Availability

The original contributions presented in the study are included in the article. Further inquiries can be directed to the corresponding author.
